# Guinea Pig as a Model to Study the Carotid Body Mediated Chronic Intermittent Hypoxia Effects

**DOI:** 10.3389/fphys.2018.00694

**Published:** 2018-06-05

**Authors:** Inmaculada Docio, Elena Olea, Jesus Prieto-LLoret, Teresa Gallego-Martin, Ana Obeso, Angela Gomez-Niño, Asuncion Rocher

**Affiliations:** ^1^Departamento de Bioquímica y Biología Molecular y Fisiología, Universidad de Valladolid, Valladolid, Spain; ^2^Instituto de Biología y Genética Molecular, Consejo Superior de Investigaciones Científicas, Universidad de Valladolid, Valladolid, Spain; ^3^CIBER de Enfermedades Respiratorias, Instituto de Salud Carlos III, Madrid, Spain; ^4^Departamento de Enfermería, Universidad de Valladolid, Valladolid, Spain; ^5^Departamento de Biología Celular, Histología y Farmacología, Universidad de Valladolid, Valladolid, Spain

**Keywords:** guinea pig, oxygen sensing, carotid body, chronic intermittent hypoxia, sympathetic activity, ventilation

## Abstract

Clinical and experimental evidence indicates a positive correlation between chronic intermittent hypoxia (CIH), increased carotid body (CB) chemosensitivity, enhanced sympatho-respiratory coupling and arterial hypertension and cardiovascular disease. Several groups have reported that both the afferent and efferent arms of the CB chemo-reflex are enhanced in CIH animal models through the oscillatory CB activation by recurrent hypoxia/reoxygenation episodes. Accordingly, CB ablation or denervation results in the reduction of these effects. To date, no studies have determined the effects of CIH treatment in chemo-reflex sensitization in guinea pig, a rodent with a hypofunctional CB and lacking ventilatory responses to hypoxia. We hypothesized that the lack of CB hypoxia response in guinea pig would suppress chemo-reflex sensitization and thereby would attenuate or eliminate respiratory, sympathetic and cardiovascular effects of CIH treatment. The main purpose of this study was to assess if guinea pig CB undergoes overactivation by CIH and to correlate CIH effects on CB chemoreceptors with cardiovascular and respiratory responses to hypoxia. We measured CB secretory activity, ventilatory parameters, systemic arterial pressure and sympathetic activity, basal and in response to acute hypoxia in two groups of animals: control and 30 days CIH exposed male guinea pigs. Our results indicated that CIH guinea pig CB lacks activity elicited by acute hypoxia measured as catecholamine (CA) secretory response or intracellular calcium transients. Plethysmography data showed that only severe hypoxia (7% O_2_) and hypercapnia (5% CO_2_) induced a significant increased ventilatory response in CIH animals, together with higher oxygen consumption. Therefore, CIH exposure blunted hyperventilation to hypoxia and hypercapnia normalized to oxygen consumption. Increase in plasma CA and superior cervical ganglion CA content was found, implying a CIH induced sympathetic hyperactivity. CIH promoted cardiovascular adjustments by increasing heart rate and mean arterial blood pressure without cardiac ventricle hypertrophy. In conclusion, CIH does not sensitize CB chemoreceptor response to hypoxia but promotes cardiovascular adjustments probably not mediated by the CB. Guinea pigs could represent an interesting model to elucidate the mechanisms that underlie the long-term effects of CIH exposure to provide evidence for the role of the CB mediating pathological effects in sleep apnea diseases.

## Introduction

For the last two decades, chronic intermittent hypoxia (CIH) has been considered a paradigm of detrimental stimulus that leads to a number of comorbidities including autonomic, cardiovascular, metabolic and cognitive dysfunction (Dempsey et al., 2010). CIH is recognized as the main hallmark in obstructive sleep apnea (OSA). It has been reported that the hypoxia/reoxygenation and oxyhemoglobin desaturation associated with apnea produces oxidative stress, inflammation and sympathetic hyperactivity, generating endothelial dysfunction and secondary systemic hypertension (Dempsey et al., 2010; Iturriaga et al., 2017).

Carotid body (CB), the main arterial oxygen sensor, triggers reflex homeostatic adjustments to acute hypoxia and is also responsible for the acclimation to high altitude. Furthermore, recent clinical and experimental evidence suggests a positive correlation between CIH, increased CB responsiveness, enhanced sympatho-respiratory coupling and arterial hypertension and cardiovascular disease (Semenza and Prabhakar, 2015; Iturriaga et al., 2017). CB type I cells are specialized in rapid response to decreased oxygen in blood. Although cellular mechanisms underlying chemo-reflexes activation by hypoxia remain to be elucidated, it has been proposed that the repeated CB type I cells stimulation produced by CIH would induce CB sensitization, increasing secretory response and chemoreceptor input to the brainstem. It originates an exaggerated sympathetic reflex that promotes a rise of circulating catecholamine (CA) and finally, hypertension (Dempsey et al., 2010). Recent evidence points to oxidative stress as the key mediator of both the enhanced CB chemosensory response to hypoxia and the hypertension induced by CIH (Iturriaga et al., 2015; Semenza and Prabhakar, 2015).

Rats and cats exposed to several days of CIH show the phenotype developed in OSA patients such as increased arterial blood pressure, hematocrit and hemoglobin and left ventricular hypertrophy, (Fletcher et al., 1992; Rey et al., 2004). Disrupting the chemo-reflex pathway by bilateral section of the carotid sinus nerve (Fletcher et al., 1992; Lesske et al., 1997) or by selective ablation of the CB while preserving the carotid baroreceptor function (Peng et al., 2014), prevented the increase of plasma CA and hypertension in CIH treated rats (Nanduri et al., 2015; Del Rio et al., 2016; Iturriaga, 2017). These findings suggest that CIH directly distresses CB function, and that other effects on brainstem neurons could be mediated by the altered sensory input from CB. Nevertheless, therapeutic removal of CB in OSA patients can have adverse consequences, such as the loss of adaptation to high altitude, the maintenance of arterial blood gases during exercise, and the cardiorespiratory responses to acute hypoxia (Prabhakar, 2016).

Most hypertension research is conducted with rodents because of blood pressure control and cardiovascular response similarities to humans. Unlike other rodents, guinea pigs show a very poor ventilatory response to hypoxia while maintaining response to hypercapnia (Schwenke et al., 2007). Recently, we have reported that guinea pig CB has a small percentage of tyrosine hydroxylase positive type I cells and they lack O_2_-sensitive K^+^ channels, which would be inhibited by hypoxia, and therefore, lacks hypoxia depolarization capacity and chemo-reflexes activation. It has also been reported that chronic sustained hypoxia (CSH) during 15 days does not modify CB activity (Gonzalez-Obeso et al., 2017). However, to date, the functionality of guinea pig CB exposed to CIH treatment has not been investigated, so we aimed to examine the long-lasting this treatment on guinea pig CB chemosensory activity, sympathetic output and its cardiorespiratory consequences. If guinea pig CB is not activated by hypoxia, CIH would not induce chemo-reflex sensitization and pathological effects derived from the CB hyperactivity would not be observed (Del Rio et al., 2016; Iturriaga, 2017). Adopting an integrative approach combining studies *in vivo* with experiments in isolated CB, we tested the hypothesis that the systemic arterial hypertensive and sympathetic hyperactivity response to CIH in guinea pig relative to other rodents would be diminished due to the CB hypofunctionality. Accordingly, we have analyzed the CB functionality, sympathetic activity and the cardiopulmonary responses to hypoxia after 30 days of CIH exposure. The ultimate purpose of this study was to determine if guinea pigs could be a model to disclose the CB dependent and non-dependent CIH effects.

## Materials and Methods

All experiments were carried out in compliance with the international laws and policies [European Union Directive for Protection of Vertebrates Used for Experimental and Other Scientific Ends (2010/63/EU)], and were reviewed and approved by the University of Valladolid Institutional Committee for Animal Care and Use.

### Animals

Experiments were performed on adult male Hartley guinea pigs (3–6 months old) with free access to standard chow and water and maintained under controlled conditions of temperature, humidity and a stationary 12 h light–dark cycle. Animals were randomly distributed in two groups: control (C) and CIH treated (21% O_2_ -80 s/5% O_2_ - 40 s; 8 h/day; 30 days), as previously described (Quintero et al., 2016). At the end of experiments, animals were euthanized by the administration of a cardiac lethal dose of sodium pentobarbital.

### Plethysmography

Ventilatory parameters as tidal volume (TV; mL/Kg), respiratory frequency (RF; breaths/min), minute ventilation (MV; mL/min/Kg) and O_2_ consumption (VO_2_; mL/min/Kg) were obtained using whole-body, unrestrained plethysmography. Methacrylate-walled chambers (Emka Technologies, Paris, France; BUXCO Research Systems, Wilmington, NC, United States) continuously fluxed (1.5 L/min) with air, hypoxic gas mixtures (12, 10, and 7% O_2_, reminder nitrogen) and hypercapnic gas mixture (5% CO_2_ in air) were used as described in Gonzalez-Obeso et al., 2017. Animals breathed air until achieving a standard resting behavior. Temperature inside the chamber was maintained within the thermo-neutral range (22–24°C) and animal temperature was constant during the experiment. Pressure modifications inside the chamber reflecting TV were calculated with a high-gain differential pressure transducer. Amplitude of pressure fluctuations is proportionally correlated to TV; a calibration of the system by injections of 5 mL air into the chamber allowed a direct estimation of TV.

The VO_2_ was measured using a CO_2_ gas analyzer (AUT4499 and BUXCO MAX II preamplifier; Buxco Research Systems). The calibration required one gas without CO_2_ and another containing a known concentration of CO_2_ (see Lighton, 2008). All parameters were recorded and analyzed with FinePointe software (Buxco Research Systems). For oximetry monitoring during guinea pig CIH exposure, multiple arterial blood samples were analyzed showing that PO_2_ nadir level was 27 mmHg (SaO_2_ ≈ 50%) in this system.

### Arterial Blood Pressure Measurement

Systolic blood pressure (SBP), diastolic blood pressure (DBP), mean arterial blood pressure (MABP), and heart rate (HR) were recorded from anesthetized animals (Ketamine 100 mg/Kg and diazepam 2 mg/Kg; i.p.) placed in supine position on a dissection table, tracheostomized and ventilated with room air (CL Palmer) (60 cycles. min^-1^ and a positive end-expiratory pressure of 2 cm H_2_O) or with the selected gas mixture (10% O_2_ and 90% N_2_; 3 min). Arterial blood pressure was continuously monitored by a catheter located in the right common carotid artery and connected to a pressure transducer (Transpac IV; ICU Medical, San Clemente, CA, United States). Signals were stored (BIOPAC Systems, Inc. MP 150, Goleta, CA; Acknowledge 3.9.1) for later analysis.

### Acid-Base Status and Blood Gases

Small arterial blood samples (0.3 ml) taken in heparinized syringes were obtained at 15 min of the baseline and after 3 min of the hypoxic challenge. Arterial pH, PO_2_, PCO_2_, HCO_3_^-^, percentage of hemoglobin saturation (SaO_2_) and hematocrit were measured (ABL 5, Radiometer Medical A/S, Copenhagen, Denmark). Erythropoietin (EPO) was measured by a commercial ELISA kit (MyBiosource, San Diego, CA, United States).

### CB Morphology and Tyrosine Hydroxylase Immunostaining

Control and CIH guinea pig CB were perfused and fixed as described in Gonzalez-Obeso et al. (2017). CB were cryoprotected by immersion in 30% (w/v) sucrose in phosphate buffer, embedded individually in Tissue-Tek^®^ (Sakura Finetek, Zoeterwoude, Netherlands) and frozen at -20°C. Tissue sections of 10 μm (Shandon Cryotome, Thermo, Electron Corporation) were collected in glass slices coated with poly L-lysine. CB sections were washed with PBS at room temperature, hematoxylin and eosin stained (H&E, Sigma-Aldrich, MO, United States), dehydrated and mounted with Eukitt Mounting Medium (Merck). Tyrosine hydroxylase (TH) immunofluorescence staining was performed in slices from control and CIH CB, identifying cell nuclei with DAPI. Sections were examined with microscope (Axioscop 2, Zeiss). Images were captured using a digital camera (CoolSNAP, Photometric, Roper Scientific) coupled to the microscope and analyzed using Metamorph 6.3 software.

Dissociated CB cells (Gomez-Niño et al., 2009) from four different control and four different CIH animals plated on several poly L-lysine-coated coverslips were immunostained for TH and nuclei with DAPI as previously described (Caceres et al., 2007; Gonzalez-Obeso et al., 2017). Cells were imaged using a laser confocal microscope (LEICA TCS, SP5) and confocal micrographs were processed using LAS software.

### Endogenous Catecholamine Content and Catecholamine Outflow From Adrenal Medulla

Endogenous CA content, in CB; superior cervical ganglion, SCG; renal artery, RA; and adrenal medulla, AM, were analyzed after organs were removed from anesthetized animals, glass to glass homogenized (0.1N perchloric acid; PCA and 0.1 mM EDTA), centrifuged and processed for HPLC analysis as described in Gonzalez-Obeso et al. (2017).

For CA outflow measurement, AM were placed in a Lucite chamber containing ice-cold Tyrode solution (in mM: 140 NaCl, 5 KCl, 2 CaCl_2_, 1.1 MgCl_2_, and 5 glucose; pH 7.4); tissues were dissected under microscope, transferred to a glass tube with Tyrode-NaHCO_3_ solution equilibrated with 20% O_2_-5% CO_2_- remainder N_2_ at 37°C and maintained in a shaker bath during 30 min, to recover from surgical stress. Each AM was transferred to vials containing 500 μl of Tyrode-NaHCO_3_ solution equilibrated either with 20% O_2_ - 5% CO_2_ - N_2_ (normoxic solution) or with 2% O_2_ - 5% CO_2_ - N_2_ (hypoxic solution). Superfusion media were collected every 10 min in eppendorf tubes containing 200 μl of 0.4N PCA, 0.1 mM EDTA and frozen at -80°C until the HPLC-ED analysis of CA was performed.

### Stimuli–Evoked Catecholamine Release From CB and Renal Artery Catecholamine Synthesis

Isolated CB were incubated 2 h with ^3^H-tyrosine of high specific activity (40–50 Ci/mmol) and the cofactors for TH and dopamine beta hydroxylase, 100 μM 6-methyl-tetrahydropterine and 1 mM ascorbic acid, respectively, to evaluate stimuli-evoked secretory response. Afterward, CB were transferred to vials containing Tyrode-bicarbonate solution (in mM: 116 NaCl, 5 KCl, 2 CaCl_2_, 1.1 MgCl_2_, 10 HEPES, 5 glucose, 24 NaCO_3_H) equilibrated with different gas mixtures containing 21 or 2% O_2_ and 5% CO_2_ (pH 7.40). High K^+^ solution was obtained by removing an equimolar amount of NaCl. Incubating solutions were changed every 10 min and their ^3^H-CA content was measured by scintillation counter as described before (Chen et al., 1997).

Renal arteries were incubated (37°C; 2 h) in Tyrode solution, containing 30 μM of 3,5-^3^H-tyrosine (6 Ci/mmol; Perkin Elmer, Boston, MA, United States), as described above for CB synthesis. After washing the tissues in precursor-free Tyrode solution (4°C; 5 min), they were homogenized and processed for HPLC-ED analysis. General procedures have been previously described (Olea et al., 2014).

### Chemoreceptor Cell Culture and Intracellular Ca^2+^ Recording

Enzymatically dispersed and dissociated CB cells were plated on poly L-lysine-coated coverslips maintained in culture for up to 24 h as formerly described (Gomez-Niño et al., 2009). Coverslips containing chemoreceptor cells were loaded with fura-2 (10 μM; Pluronic F-127; Thermo Fished Molecular Probes; 20°C, 1 h), placed in a perfusion chamber on the stage of a Nikon Diaphot 300 inverted microscope and superfused with Tyrode solution. Fura-2 is excited at λ = 340 nm and 380 nm and emits fluorescence at 540 nm, collected with a SensiCam digital Camera (PCO CCD imaging, PCO, Kelheim, Germany). The background was removed (MetaFluor program, Molecular Devices, Wokingham, United Kingdom) and the variations in the cytosolic Ca^2+^ were presented as the fluorescence emitted after excitation at 340 nm and the fluorescence emitted after excitation at 380 nm (ratio 340 nm/380 nm). The illumination system and camera were driven by Axon Imaging Workbench 4.0 (Molecular Devices, Wokingham, United Kingdom) running on a Pentium computer. Hypoxia (5% CO_2_, 95% N_2_) and 35 mM KCl were used as stimuli.

### Data Presentation and Statistical Analysis

Results are presented as mean ± SEM. Statistical analyses were completed by GraphPad Prism version 6.0. Mean value comparisons were performed with unpaired Student’s *t*-test, One-Way Analysis of Variance (ANOVA) with Dunnett’s multiple comparison tests, and by Two-way ANOVA with Tukey’s or Sidak’s multi-comparison test, according to the structure of data. A *p-*value < 0.05 was considered as statistically significant. All comparisons of experimental data were performed with two-tailed tests.

## Results

### Ventilatory Response

**FIGURE 1:**
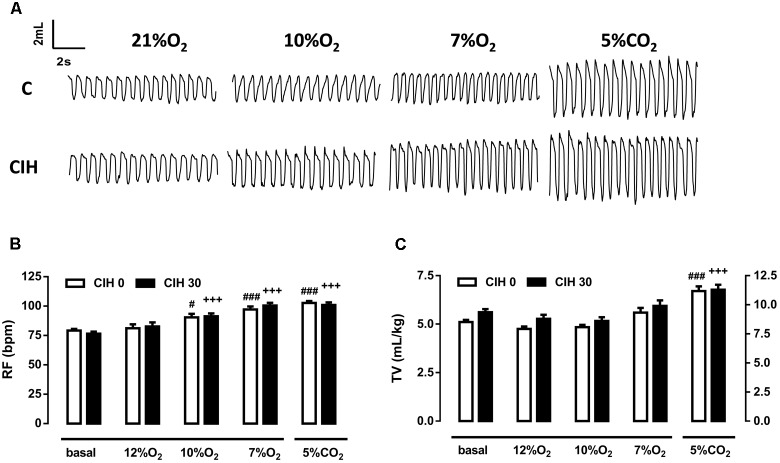
Effect of 30 days of chronic intermittent hypoxia (CIH30) on guinea pig breathing pattern. **(A)** Sample plethysmography recordings of one control (CIH0) and one CIH30 guinea pig breathing air (21% O_2_), or 10% O_2_, 7% O_2_ and 5% CO_2_ acute tests. In **(B)**, Respiratory frequency (RF) expressed as breaths per minute (bpm) and in **(C)**, Tidal volume (TV) expressed as mL/kg from CIH0 and CIH30 guinea pigs in response to acute hypoxia (12% O_2_, 10% O_2_, 7% O_2_) and hypercapnia (5% CO_2_) tests. #*p* < 0.05; ##*p* < 0.01; ###*p* < 0.001 vs. basal CIH0; +++*p* < 0.001 vs. basal CIH30. Data are mean ± SEM; *n* = 16. Two-way ANOVA with Tukey’s multiple comparison test for analysis between CIH0 and CIH30, and one-way ANOVA with Dunnett’s multiple comparison test for analysis intra group (vs. basal).

**FIGURE 2:**
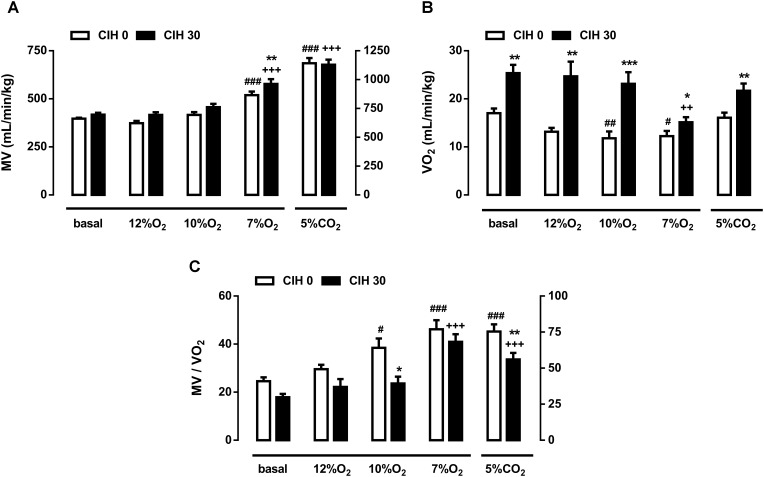
Effect of chronic intermittent hypoxia on guinea pig ventilation. **(A)** Minute volume (MV; mL/min/Kg) from guinea pigs breathing air, different acute hypoxia (12% O_2_, 10% O_2_, and 7% O_2_) and hypercapnia (5% CO_2_) tests. ###*p* < 0.001 vs. basal CIH0; +++*p* < 0.001 vs. basal CIH30; ^∗∗^*p* < 0.01 CIH0 vs. CIH30. **(B)** Oxygen consumption (VO_2_; mL/min/kg) of CIH0 and CIH30 guinea pigs in response to the different gas mixtures. #*p* < 0.05 ##*p* < 0.01 vs. basal CIH0; ++*p* < 0.01 vs. basal CIH30; ^∗∗^*p* < 0.01 ^∗∗∗^*p* < 0.001 CIH0 vs. CIH30. **(C)** Normalized ventilation to oxygen consumption (MV/VO_2_) calculated from guinea pigs breathing under the same conditions described above. #*p* < 0.05; ###*p* < 0.001 vs. basal CIH0; +++*p* < 0.001 vs. basal CIH30; ^∗^*p* < 0.05 ^∗∗^*p* < 0.01 CIH0 vs. CIH30. Data are mean ± SEM; *n* = 16. Two-way ANOVA with Tukey’s multiple comparison test for analysis between CIH0 and CIH30 groups, and one-way ANOVA with Dunnett’s multiple comparison test for analysis intra group (vs. basal).

Ventilatory response to acute hypoxic tests (12, 10, and 7% O_2_; 10 min) and hypercapnic test (5% CO_2_; 10 min) were assessed by continuous recording of respiratory frequency (RF), tidal volume (TV), and minute volume (MV) in the same animals at two different times: initially, before intermittent hypoxia exposure (CIH0), and after 30 days of intermittent hypoxia treatment (CIH30). **Figure 1A** depicts single respiratory recordings obtained from one animal breathing each of the different atmospheres. Ventilatory parameters from CIH0 breathing room air were not modified when exposed to 12% O_2_ but frequency significantly increased when guinea pigs breathed 10 and 7% O_2_ (*p* < 0.05 and 0.001, respectively). CIH30 behaved similarly (*p* < 0.001 in both cases), as shown in **Figure 1B**. TV was not modified at any hypoxic tests in CIH0 or CIH30 (**Figure 1C**). Hypercapnic mixture (5% CO_2_) produced the same significant increase of RF and TV in CIH0 and CIH30 animals (*p* < 0.001; **Figures 1B,C**). The effect of 30 days of CIH exposure (CIH0 vs. CIH30) produced a slight (no statistically significant) increase of RF and TV in animals breathing 7% O_2_ (**Figures 1B,C**). However, when the MV was calculated (see **Figure 2A**) significant differences were found (*p* < 0.01 CIH0 vs. CIH30).

**Figure 2A** shows the MV obtained in guinea pigs breathing different gas mixtures. Ventilation in normoxia, 12 and 10% O_2_ were unaffected by exposure to CIH, but significantly increased when animals breathed 7% O_2_ in CIH0 and CIH30: 397 ± 5 breathing room air and 519 ± 19 ml/min/kg breathing 7% O_2_ in CIH0 guinea pigs vs. 418 ± 10 breathing room air and 578 ± 25 ml/min/kg breathing 7% O_2_ in CIH30 (*p* < 0.001). Furthermore, MV increase at 7% O_2_ hypoxia test was significantly higher in CIH30 than CIH0 guinea pigs (*p* < 0.01). Acute hypercapnia test produced a similar significant MV rise (1141 ± 45 in CIH0 vs. 1129 ± 43 ml/min/kg in CIH30; *p* < 0.001) compared with respective baseline values. Ventilatory response to Dejours test (100% O_2_; 3 min) showed no ventilatory differences between CIH0 and CIH30 (data not shown). **Figure 2B** represents the oxygen consumption (VO_2_) under the conditions described above. CIH0 animals breathing 10 and 7% O_2_ atmosphere showed significantly metabolism decrease (17.1 ± 1.0 breathing air, 11.8 ± 1.4 breathing 10% O_2_ and 12.2 ± 1.1 mL/min/kg breathing 7% O_2_) compared to the baseline value. Nonetheless, CIH30 guinea pigs significantly increased VO_2_ breathing room air, 12 and 10% O_2_, except when breathing 7% O_2_ or 5% CO_2_ atmospheres, in which there were no differences between groups. **Figure 2C** depicts the ventilation standardized by metabolic rate (MV/VO_2_) showing hypoxic hyperventilation at 10 and 7% O_2_ (*p* < 0.01), and hypercapnic hyperventilation (*p* < 0.001) in CIH0. Conversely, CIH30 group only hyperventilated at 7% O_2_ and 5% CO_2_ (*p* < 0.001). Consequently, CIH30 exposure blunted the hyperventilatory response at 10% O_2_ (*p* < 0.05) and at hypercapnia (*p* < 0.01).

### Acid-Base Status and Blood Parameters

**Table 1** shows blood values for pO_2_, pCO_2_, pH, HCO_3_^-^, and SaO_2_ obtained from small arterial blood samples taken in normoxia (breathing air) or after acute hypoxia (10% O_2_; 3 min) in C and CIH guinea pigs. All parameters were comparable when guinea pigs breathed air in C and CIH groups. Neither differences found between groups, except for the lower SaO_2_ after the acute hypoxia test in CIH guinea pigs (44 ± 6 vs. 62 ± 5%, respectively; *p* < 0.05). Whereas hematocrit was identical (41.5 ± 0.8 vs. 41.4 ± 0.8%) in both groups, EPO increased in CIH animals (214 ± 52 vs. 141 ± 17 mU/mL; *p* < 0.05).

**Table 1:** Arterial blood gasometry data, hematocrit and erythropoietin measurements in control and CIH guinea pigs breathing normoxia (21% O_2_) or acute hypoxia (10% O_2_).

Guinea pigs	C		CIH	
		
	21% O_2_	10% O_2_	21% O_2_	10% O_2_
pO_2_(mmHg)	65 ± 6	29 ± 3^+++^	64 ± 6	22 ± 2^+++^
pCO_2_(mmHg)	36 ± 2	32 ± 1	38 ± 3	36 ± 2
PH	7.49 ± 0.02	7.52 ± 0.01	7.47 ± 0.02	7.49 ± 0.01
HCO_3_^-^ (mM)	27 ± 1	26 ± 1	27 ± 1	27 ± 1
SaO_2_ (%)	91 ± 2	62 ± 5^+++^	90 ± 3	44 ± 6^+++^^∗^
Ht (%)	41.5 ± 0.8		41.4 ± 0.8	
EPO (mU/mL)	141 ± 17		214 ± 52^∗^	


*+++*p* < 0.001 10% O_2_ vs. 21% O_2_ in each group; ^∗^*p* < 0.05 CIH vs. C. Data are mean ± SEM (*n* = 8). Two-Way ANOVA with Sidak’s multiple comparison test.*

### CB Morphology and Stimuli-Dependent Activation

At day 0 guinea pigs body weight was 637 ± 14 g in C and 610 ± 17 g in CIH0 (*p* > 0.05; unpaired *t*-test). After 30 days, body weight was 802 ± 19 g in C and 709 ± 14 g in CIH guinea pigs (*n* = 16, *p* < 0.01 unpaired *t*-test). However, CB weight was no different in C and CIH animals (81 ± 4 μg vs. 87 ± 5 μg, respectively; *p* > 0.05; *n* = 8).

**FIGURE 3:**
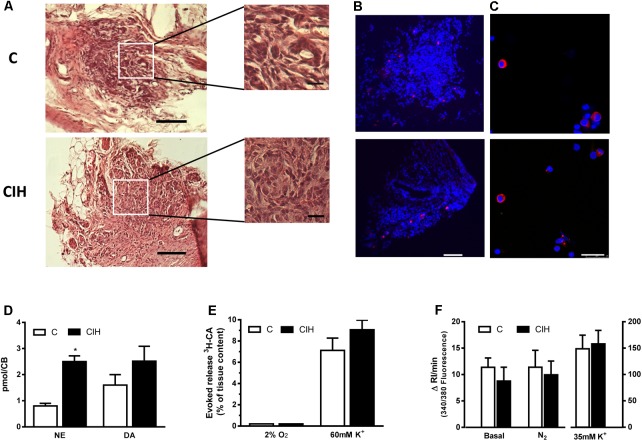
CB morphology and stimuli-dependent response from control and CIH guinea pig. **(A)** Hematoxylin and eosin staining of a guinea pig CB section (control on top, CIH on bottom); calibration bar 100 μm (20 μm in inserts). **(B)** Immunohistochemical tyrosine hydroxylase (TH; red) and DAPI (blue) cell identification from slices of control (top) and CIH (bottom) CB; calibration bar 100 μm. **(C)** Immunocytochemical TH (red) and DAPI (blue) identification of isolated control (top) and CIH (bottom) guinea pig CB cells. Calibration bar 25 μm. **(D)** Endogenous content of norepinephrine (NE) and dopamine (DA) expressed as pmol/CB in control and CIH animals measured by HPLC-ED; ^∗^*p* < 0.05 CIH vs. C; Data are mean ± SEM (*n* = 8–12); Two-way ANOVA with Sidak’s multiple comparison test. **(E)** Evoked release of ^3^H-CA from control and CIH CB in response to 2%O_2_ or high-external K^+^ (60 mM). Data are mean ± SEM (*n* = 6); Two-way ANOVA with Sidak’s multiple comparison test. **(F)** Mean intracellular calcium levels, expressed as the increase in the integrated response per minute, in control and CIH chemoreceptor cells under basal conditions and in response to severe hypoxia (5% CO_2_/95% N_2_) or 35 mM K^+^. Data are mean ± SEM (*n* = 28–33); Two-way ANOVA with Sidak’s multiple comparison test.

**Figure 3A** shows the whole surface of 10 μm thick slices from control (top) and CIH (bottom) guinea pig CB, stained with hematoxylin and eosin. An extended detail of the parenchyma from each slice is also shown. No substantial differences in the structure of the tissue were observed. **Figure 3B** shows TH immunostaining in CB slices from control and CIH animals. The TH positive staining area was lower than that observed in CB from other rodents. **Figure 3C** shows chemoreceptor cells dissociated from CB cultured and immunostained for TH from C and CIH animals. The percentage of TH-positive type I cells was 7% in both cases (by cumulative counting of several cultures), obtained from 4 guinea pigs of each group, a percentage also lower than that observed in CB from other rodents.

**Figure 3D** shows the endogenous CA content in CB from C and CIH animals. NE content was significantly higher in CIH animals (*p* < 0.05) and although DA content was slightly higher in CIH, the increase was not statistically significant. **Figures 3E,F** show the lack of response to hypoxia from *in vitro* CB, expressed as evoked CA secretion (% of tissue content) or Ca_i_^2+^ changes, in both groups of animals. Unlike this lack of response to hypoxic stimulus, CB from both groups released comparable amounts of ^3^H-CA (7.1 ± 1.1% vs. 9.0 ± 0.9%, respectively; *p* > 0.05; **Figure 3E**), and increased to comparable rise of Ca_i_^2+^ in response to high external K^+^ (**Figure 3F**).

### Endogenous Catecholamine Content

**FIGURE 4:**
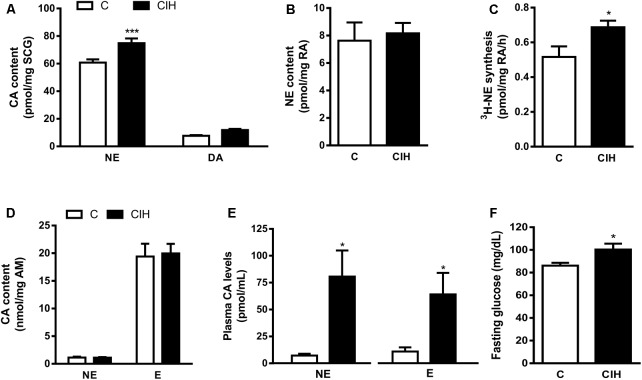
Sympathetic activity in tissues from control and CIH guinea pigs. **(A)** Superior cervical ganglion (SCG) endogenous content of norepinephrine (NE) and dopamine (DA) expressed as pmol/mg tissue in control and CIH measured by HPLC-ED. ^∗∗∗^*p* < 0.0001 CIH vs. C. Data are mean ± SEM (*n* = 14–16). Two-way ANOVA with Sidak’s multiple comparison test. **(B)** Renal artery (RA) endogenous NE levels expressed as pmol/mg tissue. Data are mean ± SEM (*n* = 6–7); Unpaired *t*-test. **(C)** Rate of ^3^H-NE synthesis expressed as pmol/mg tissue/h in RA. ^∗^*p* < 0.05 CIH vs. C. Data are mean ± SEM (*n* = 6–7); Unpaired *t*-test. **(D)** Adrenal medulla (AM) endogenous content of NE and epinephrine **(E)** expressed as nmol/mg tissue. Data are mean ± SEM (*n* = 14–16); Two-way ANOVA with Sidak’s multiple comparison test. (E) Plasma NE and **(E)** levels expressed as pmol/mL. ^∗^*p* < 0.05 CIH vs. C; Data are mean ± SEM (*n* = 6–8); Unpaired *t*-test. **(F)** Fasting glucose plasma levels expressed as mg/dL. ^∗^*p* < 0.05 CIH vs. C; Data are mean ± SEM (*n* = 16); Unpaired *t*-test.

The lack of effect of CIH exposure on the CB activity prompted the analysis of the CA content in sympathetic endings of RA, SCG, AM and plasma CA levels as an index of sympathetic activity. Selective differences were found in tissue CA content from SCG and plasma CA levels. As shown in **Figure 4A**, NE content was 60.8 ± 2.3 in C and 74.8 ± 3.4 pmol/mg SCG in CIH (*n* = 12; *p* < 0.001); DA content was 7.7 ± 0.5 in C and 11.8 ± 0.8 pmol/mg SCG in CIH group. There were no changes in RA and AM endogenous CA content from both groups **Figure 4B** shows the similar RA content of NE in C and CIH guinea pigs (7.6 ± 1.3 pmol/mg vs. 8.2 ± 0.7 pmol/mg of tissue, respectively). However, ^3^H-NE syntheses was significantly higher in RA from CIH guinea pigs (0.52 ± 0.06 vs. 0.69 ± 0.04 pmol/mg/h of tissue in C and CIH; **Figure 4C**). Adrenal medulla CA content was also similar in both groups (1.13 ± 0.15 vs. 1.11 ± 0.01 nmol/mg of tissue of NE and 19.4 ± 2.3 vs. 20 ± 1.7 nmol/mg of tissue of E in C and CIH, respectively; **Figure 4D**). Plasma NE was significantly higher in CIH than in C guinea pigs (80.7 ± 24.3 vs. 7.2 ± 1.7 pmol/mL). Epinephrine (E) was 11 ± 4 pmol/mL in C and also significantly higher in CIH (64 ± 20 pmol/mL; *n* = 6–8, *p* < 0.05; **Figure 4E**). Fasting glucose levels also were significantly higher in CIH than in C guinea pigs (100 ± 5 vs. 86 ± 2 mg/dL; *p* < 0.05; **Figure 4F**).

### Cardiovascular Effects

**FIGURE 5:**
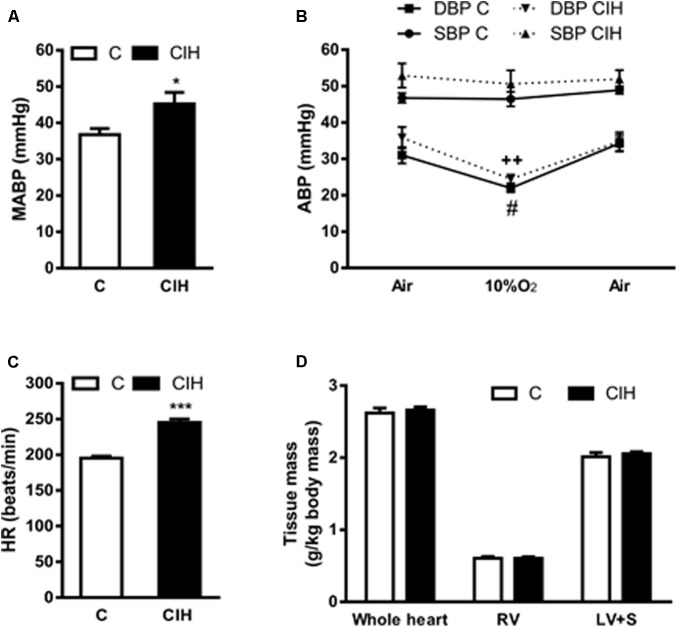
Cardiovascular parameters from control and CIH guinea pigs. **(A)** Mean arterial blood pressure (MABP) in anesthetized control and CIH guinea pigs breathing air, measured by carotid artery catheterization. ^∗^*p* < 0.05 CIH vs. C. unpaired *t*-test; Data are mean ± SEM (*n* = 7); Unpaired *t*-test. **(B)** Systolic (SBP) and diastolic (DBP) blood pressure in basal (air), acute hypoxia (10% O_2_) and recovery (air) from control and CIH guinea pigs. #*p* < 0.05, 10% O_2_ vs. air C (DBP); ++*p* < 0.01 10% O_2_ vs. air CIH (DBP); Data are mean ± SEM (*n* = 7–8); Two-way ANOVA with Tukey’s multiple comparison test. **(C)** Heart rate (beats per minute) measured in control and CIH guinea pigs breathing air. ^∗∗∗^*p* < 0.001, CIH vs. C; Data are mean ± SEM (*n* = 7); Unpaired *t*-test. **(D)** Weight of whole heart, right ventricle, and left ventricle plus septum, normalized by the body weight, expressed as g/kg body mass in control and CIH guinea pigs. Data are mean ± SEM (*n* = 7); Two-way ANOVA with Sidak’s multiple comparison test.

Consistent with the increase in plasma CA levels we found that (MABP from anesthetized guinea pigs also increased in the experimental CIH group, although the effect was less noticeable (45 ± 3 vs. 37 ± 2 mmHg in CIH and C, respectively; *p* < 0.05; **Figure 5A**). In **Figure 5B** is represented the systolic (SBP) and diastolic (DBP) blood pressure obtained from animals breathing air, acute hypoxic test (10% O_2_) and recovery (air). The profile of SBP (47 ± 1 and 46 ± 2 mmHg in C group vs. 53 ± 3 and 51 ± 4 mmHg in CIH breathing air and 10%O_2_, respectively) and DBP (31 ± 2 and 22 ± 1 mmHg in C group vs. 36 ± 3 and 24 ± 1 mmHg in CIH, breathing air and 10% O_2_, respectively) were similar in both groups of animals, but slightly higher in CIH. **Figure 5C** shows the significant HR increase in CIH guinea pigs (245 ± 5 vs. 195 ± 3 bpm; *p* < 0.001). No differences were observed in whole heart, right ventricle or left ventricle plus septum weight between C and CIH guinea pigs (**Figure 5D**).

### Catecholamine Outflow From the Adrenal Medulla

**FIGURE 6:**
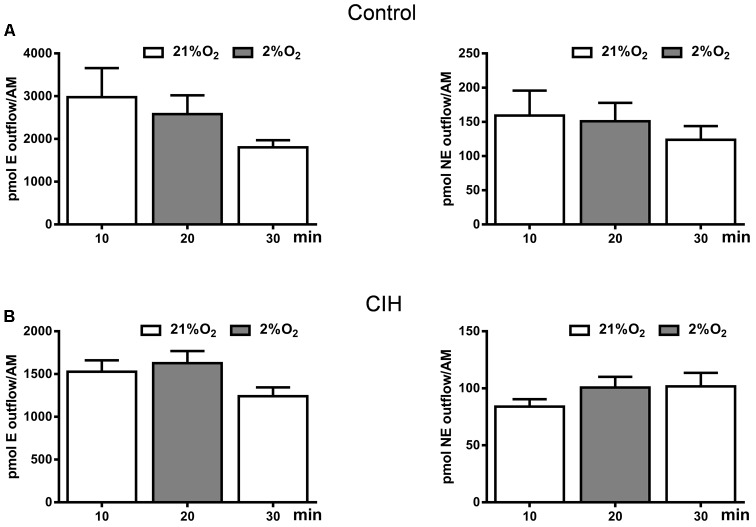
Catecholamine outflow from control and CIH guinea pig adrenal medulla. Time course of endogenous epinephrine (E; left) and norepinephrine (NE; right) outflow from control **(A)** and CIH **(B)** guinea pig AM in response to basal (21% O_2_) or hypoxia (2% O_2_) conditions, expressed as pmol/AM. Data are mean ± SEM (*n* = 4–6); One-way ANOVA with Tukey’s multiple comparison test.

Data presented in **Figure 4D** have not underscored a clear differential behavior in the adrenal medulla CA content after CIH exposure. However, increase in plasma CA levels (**Figure 4E**) and metabolic response (**Figure 4F**) in CIH guinea pigs prompted checking the possible direct hypoxic sensitivity of adrenomedullary chromaffin cells. We studied the effect of hypoxia (2% O_2_) on *in vitro* adrenal medulla CA outflow from both groups. Results presented in **Figure 6** show that hypoxia does not elicit CA efflux, not altering the ongoing time course of the outflow from AM in either group. In normoxic conditions (21% O_2_), the amount of E in the incubation solution was 2979 ± 678 pmol/AM and hypoxia (2% O_2_) induced an outflow of 2583 ± 439 pmol/AM in C guinea pigs. NE levels were 159 ± 36 pmol/AM in normoxia and 151 ± 26 pmol/AM in hypoxia in the same group. Under the same conditions, the amount of E in the AM effluent from CIH guinea pigs was 1527 ± 133 pmol/AM in 21% O_2_ and 1628 ± 140 pmol/AM in 2% O_2_. NE values were 84 ± 6 and 100 ± 9 pmol/AM in normoxia and hypoxia, respectively, from CIH animals. Data showed that CIH does not provide hypoxia-sensitivity to guinea pig AM.

## Discussion

The main results of this work are the lack of CB sensitization after CIH exposure during 30 days in this species and, as a consequence, the lack of respiratory reflex responsiveness to acute hypoxia (except to 7% O_2_), which has been described in rodents with hypoxia sensitive CB. These findings extend previous observations on the effect of acute and CSH treatment on CB responses in guinea pigs (Gonzalez-Obeso et al., 2017). From an integrative approach, combining functional studies *in vivo* and *in vitro*, we have tested the hypothesis that the systemic arterial hypertensive effect of CIH would be removed or ameliorated by the lack of CB responsiveness in guinea pigs. Our data partially support the hypothesis tested, showing that guinea pigs, compared to rats, have a small systemic arterial pressure change after CIH, despite similar sympathetic effects, suggesting a critical role for CIH induced sensitization in oxygen sensing mechanisms in the CB. Conversely to this species, it has been reported that adult rats exposed to 10 days of CIH have augmented hypoxic sensory response (Peng and Prabhakar, 2004) and a similar effect was also observed in cats (Rey et al., 2004) and mice (Peng et al., 2006). Moreover, rats exposed to 30 days of CIH develop hypertension and increased sympathetic nerve activity; these responses are prevented by chronic bilateral sectioning of carotid sinus nerve (Lesske et al., 1997). Our findings constitute the first data of CIH effects on guinea pig ventilation, CB functionality and sympatho-adrenal activation.

Previous work showing that the poor effect of acute hypoxia on ventilation correlates with the lack of CB activity suggested a lack of action potential generation in the carotid sinus nerve (Schwenke et al., 2007; Gonzalez-Obeso et al., 2017). Although we did not measure the afferent activity in the carotid sinus nerve in our preparation, it is widely accepted that afferent chemosensory activity results from synaptic activation mediated by neurotransmitters released from type I cells (Gonzalez et al., 1994; Prabhakar, 2000; Iturriaga and Alcayaga, 2004). The fact that there was no Ca_i_^2+^ increase or CA secretion from CB in response to hypoxia in CIH exposed animals, suggests that there was no sensory nerve activity in CB from CIH guinea pigs, as is the case for normoxic animals (Schwenke et al., 2007). This would be the cause for the absence of hypoxic, but not hypercapnic, ventilatory response.

Activation of chemoreceptor type I cells by hypoxia depends on the inhibition of O_2_-sensitive plasma membrane K^+^ channels, leading to cell depolarization, Ca^2+^ influx and neurotransmitter release. This model of chemoreceptor activation, accepted for several animal models, does not fit the guinea pig CB behavior. Type I cells contain DA allowing to visualize with TH antisera, the rate-limiting CA synthesis enzyme. In a previous study we analyzed the expected presence of TH and the occurrence of endogenous CA (by HPLC). We observed clear species differences between rat and guinea pig CB type I cells with respect to the immunoreactivity to TH antisera. In spite of the similar size to rat CB, a very small number of TH positive cells was found in guinea pig CB and lower levels of CA than in rat CB. We did not find serotonin in control or CIH guinea pig CB as has been reported in rat CB exposed to CIH (Peng et al., 2009). Guinea pig type I cells lack O_2_-sensitive K^+^ channels, and therefore, they would lack hypoxia depolarization capacity and CA secretory response (Gonzalez-Obeso et al., 2017). After CIH we did not observe hypoxia elicited secretory response or intracellular calcium increase, suggesting that CIH exposure does not contribute to developing hypoxia depolarization capacity in guinea pig CB type I cells.

The fact that the guinea pig CB is hypofunctional and does not respond to the full hypoxia range (mild to severe hypoxia) correlates with a blunted ventilatory response in both C and CIH guinea pigs. These breathing responses are typical of mammals and humans adapted to high altitude (Monge and León-Velarde, 1991). The absence of hypoventilation during the Dejours test indicates that, unlike rats and other species, CB is not a contributing drive to normoxic ventilation in guinea pigs. In conscious animals, only severe hypoxia caused a significant MV increase before (30%) and after (38%) CIH exposure, mainly caused by augmented respiratory frequency. Yet, the difference between both responses was significant (25%; *p* < 0.05), meaning that there was a chemo-reflex sensitization after CIH treatment. Acute hypoxia in CIH0 guinea pigs significantly decreased O_2_ consumption (breathing 10 and 7% O_2_); this would imply that a metabolic depression compensates the blunted ventilation in hypoxic CIH0 guinea pigs. Conversely, after CIH exposure there was an increase (≈50%) in oxygen consumption when breathing air, 12 or 10% O_2_. Increased O_2_ consumption in CIH animals mismatches their low ventilation. Consequently, normalizing ventilation to O_2_ consumption in both groups, we can observe hyperventilation at 10 and 7% O_2_ in CIH0 but a blunted ventilation at 10% O_2_ and an unaffected response to the most intense hypoxic stimulus (7% O_2_), after CIH exposure. Increased ventilation and decreased metabolism after CIH, renders similar values for MV/VO_2_ ration when breathing 7% O_2_ in both groups of animals. Ventilatory response to hypercapnia is similar before and after CIH treatment and comparable to that observed in the rat (Gonzalez-Obeso et al., 2017). However, it can be also observed hypoventilation when normalized to higher oxygen consumption after CIH exposure. Guinea pig CB sensory denervation decreased 28% the hyperventilation induced when breathing a hypercapnic mixture (Schwenke et al., 2007), suggesting that hyperventilation is also mediated by central (≈70%) and arterial (≈30%; Gonzalez et al., 1994) chemoreceptors.

Previously, we have observed that anesthetized guinea pigs have a considerably low (60 mmHg) arterial blood PO_2_ (Gonzalez-Obeso et al., 2017). This finding, comparable to other reported PO_2_ values of 66 to 57 mmHg in awake and anesthetized guinea pigs (Feuerstein et al., 1985; Mover-Lev et al., 1997; Schwenke and Cragg, 2004), is unaffected after CIH exposure (65 mmHg in C vs. 64 mmHg in CIH). Hypoxic challenge (10% O_2_) diminished SaO_2_ even more in CIH (44% vs. 66% in C) because of a higher metabolism (**Figure 2B**). It is known that guinea pigs have a high hemoglobin affinity (P_50_≈27 mmHg) compared to rats. This high affinity will secure lung oxygenation at low PO_2_ (Turek et al., 1980). Low PO_2_ and low SaO_2_ would be also compensated by an increase in red blood cells (RBC) and a concomitant oxygen blood transport capacity. In hypoxia, RBC rise to enhance oxygen delivery to tissues by increased erythropoiesis that is mediated by HIF-2, the main regulator of EPO gene transcription (Rankin et al., 2007). However, a rapid change of oxygen tension from hypoxia to normoxia rectifies the hypoxia-induced RBC growth, preferentially eliminating the new RBC or neocytes, a process coined as neocytolysis (Alfrey et al., 1997; Risso et al., 2007). This would be the reason that OSA patients, with significant cycles of severe hypoxia during sleep, do not present polycythaemia (Solmaz et al., 2015; Song et al., 2017). We think this also would be the reason that CIH guinea pigs have identical haematocrit to normoxic control ones, in spite of a significantly higher level of EPO (214 vs. 141 mU/mL in C); the increased EPO level in CIH group should be considered as a HIF-mediated adaptive response to CIH.

The fact that the guinea pig CB is not sensitized by CIH would strengthen our hypothesis: systemic effects related to CIH in other species should be absent in guinea pigs. However, several paradoxical results would point to an activation of the sympathetic system after CIH: (i) a slight increase in arterial blood pressure (≈10 mmHg); (ii) a slight increase in HR (≈25%); (iii) a large increase in plasma CA levels; and, (iv) an increase in renal artery CA synthesis (≈40%). Guinea pigs are hypotensive animals compared to other rodents (Schwenke and Cragg, 2004; Gonzalez-Obeso et al., 2017) which has been related to the low noradrenergic tone and to a higher capillarity in peripheral tissues decreasing peripheral resistances. The observed increase in plasma NE levels could explain the higher MABP and HR found from guinea pigs after CIH exposure. These results suggest that guinea pigs would possess an O_2_-sensing mechanism, other than the CB, responsible for the sympathetic cardio-circulatory reflex (Cardenas and Zapata, 1983; Guyenet, 2000). This mechanism remains to be studied.

The CB and AM are sympatho-adrenal tissues with the same developmental origin, functioning as a unit that originates cardiorespiratory reflexes in response to systemic hypoxia. A functional CB-AM axis is essential for hypoxic environment survival, and the most efficient response is the hyperventilation triggered by CB chemoreceptors, nearly absent in guinea pigs (Gonzalez-Obeso et al., 2017). Therefore, we envisioned the possibility that metabolic responses would be over-developed in guinea pigs as a result of the sympathetic activity and the intrinsic sensitivity of AM to hypoxia, mimicking the situation of neonatal animals (Seidler and Slotkin, 1985; Thompson et al., 1997; Nurse et al., 2017). The increased plasma CA and fasting glucose levels, and the blood pressure modifications observed in CIH guinea pigs would arise from the AM sensitization. We also found endogenous CA increase (mainly NE) in SCG, but no changes in AM content. Sympathetic nerve endings are the origin of plasma NE, and E is secreted from AM, so plasma levels of NE and E can represent an index of the general sympathetic tone (Goldstein et al., 2003; Kjaer, 2005). Plasma E/NE ratio might change under stress conditions, such as intermittent hypoxia; therefore, the different source of both CA would allow disclosing the main origin of plasma CA; an intermittent hypoxia activation of the AM would increase the E/NE ratio. However, plasma E/NE ratio was lower in CIH than in C guinea pigs, indicating that AM contribution to plasma CA is lower than sympathetic endings and CIH does not activate AM, but increases sympathetic tone. The increased NE content in SCG would reinforce this finding. We have also studied the effect of CIH on the time course of the CA outflow from isolated AM in both groups of guinea pigs. CIH exposure did not alter hypoxia elicited time course of E and NE adrenal outflow from *in vitro* AM. The lack of response of adult *in vitro* AM to hypoxia was expected from previously published findings in adrenomedullary cells from rats (Seidler and Slotkin, 1985; Thompson et al., 1997) and from our own data in guinea pigs (Olea et al., 2018). However, Prabhakar’s group reported that CIH induced oxidative stress and facilitates catecholamine efflux from the AM slices in adult rats (Kumar et al., 2015). The absence of the *in vitro* AM and CB response to hypoxia in guinea pigs leads to question how the sympatho-adrenal activation is achieved in CIH. Most of the response would be reflex-triggered by the CB in rats (Gonzalez et al., 1994; Marshall, 1994). In guinea pigs, due to the lack of functional CB, there must be additional hypoxia-sensitive structures capable of triggering the sympatho-adrenal activation. Several studies have recognized caudal hypothalamus and rostral ventrolateral medulla areas directly activated by hypoxia in different species, increasing sympathetic discharges, blood pressure and HR (Guyenet, 2000; Neubauer and Sunderram, 2004; Marina et al., 2015).

In summary, CIH exposure has no effect on the guinea pig CB, and none or poor effect on basal ventilation but blunted respiratory responses to acute hypoxic challenges. The lack of response to hypoxia suggests that CIH does not modify the excitability of CB type I cells or their synaptic communication with afferent endings. Moreover, similar time and intensity treatments in other rodents produce profound long-lasting effects in chemosensory reflex and related pathological effects. Further studies are needed to clarify the missing mechanisms that underlie the lack of long-term effects of CIH exposure on the guinea pig CB to provide evidence for the role of the CB in mediating hypertension in OSA. Therefore, guinea pigs could represent an interesting model to study the brainstem sensitivity to hypoxia, the oxygen sensing plasticity and the cardiovascular-endocrine responses elicited by chronic intermittent low PO_2_.

## Author Contributions

AG-N and AR designed the study and prepared the manuscript. ID, EO, JP-L, TG-M, AO, AG-N, and AR carried out the experiments and analyzed the data. JP-L and EO designed graphs. All authors read and approved the final manuscript.

## Conflict of Interest Statement

The authors declare that the research was conducted in the absence of any commercial or financial relationships that could be construed as a potential conflict of interest.
